# Patient choice significantly affects mastectomy rates in the treatment of breast cancer

**DOI:** 10.1186/1477-7800-5-20

**Published:** 2008-08-11

**Authors:** Robert M Kirby, Abdul Basit, Natarajan Manimaran

**Affiliations:** 1 The Breast Unit, University Hospital of North Staffordshire, Stoke on Trent, Staffordshire, ST4 6QG, UK; 2 Department of Surgery, University of Keele, Keele, Staffordshire, UK

## Abstract

Mastectomy rates may be affected by patient choice. 203 patients who had a Total Mastectomy for breast cancer were invited to complete questionnaires at routine follow up clinics to ascertain if they had been offered a choice of Breast Conserving Surgery (BCS), and to establish the reasons for their preference. Questionnaires were checked against medical and nursing records to confirm the reasons for the patients' choice of mastectomy. 130 patients (64%) chose to have a mastectomy, reporting that they felt safer (n = 119); wanted to decrease the risk of further surgery (n = 87) and/or wished to avoid radiotherapy (n = 34). Some were advised not to have BCS if they had a large tumour size, central or multifocal tumours and/or associated extensive microcalcification on mammography (n = 29). 24 patients had BCS as first operation but had repeat surgery for involved or narrow excision margins. Despite being advised that there is no difference between survival rates of this and breast conserving surgery, many patients still feel safer with mastectomy.

## Introduction

The NHS breast screening service monitors mastectomy rates across individual regions and individual Trusts. Breast Conserving Surgery is an appropriate choice for the surgical treatment of early stage breast cancer and when combined with postoperative radiotherapy to the remaining breast tissue it provides survival equivalent to total mastectomy [1]. The reasons for variation in mastectomy rates between different units remain a matter of conjecture and debate. Although patient choice is usually the quoted reason for some units having a higher rate, this is rarely tested.

The North Staffordshire Breast Screening Unit had a 34.4% mastectomy rate for women diagnosed with breast cancer during the period 2000–2004. This compares to a national mastectomy rate of 27.8% in the United Kingdom and a 28.6% mastectomy rate for the West Midlands region (P = 0.075) [2]. By implication, units with a higher mastectomy rate may be thought to present their patients with fewer alternatives. This study was designed to determine the influence of patient choice on the North Staffordshire mastectomy rate.

## Background

North Staffordshire has a population of 234,268 females. Breast screening commenced in North Staffordshire in 1988 [3]. The population of North Staffordshire is recognised as being one of the poorer populations within Britain. 60–70% of the resident population of North Staffordshire forms the bottom quintal in England in terms of life expectancy. Representation in higher wage sectors is only around half the average of Great Britain and 60% of the working age population in Stoke-on-Trent, the biggest city in North Staffordshire, possesses no qualifications or else is only qualified to NVQ1 compared to the British average of 47% [4].

## Methods

A questionnaire was administered to mastectomy patients returning for routine follow up examination in the out-patient clinic. All patients were under the care of a single consultant (RMK) having been treated for either screen detected cancer or for symptomatic disease. The overall mastectomy rate for patients within this combined group was 52% of 1251 patients. Overall 115 patients had re-operative surgery following wide local excision for margin involvement for close margins of less than 5 mm in invasive carcinoma and less than 10 mm in Ductal carcinoma in-situ, or for involved cavity biopsies.

All mastectomy patients were offered questionnaires by clinic nursing staff. Medical and breast care nursing notes were reviewed for each patient and the histology of each patient who has completed the questionnaire was checked. Patients were asked whether they had been offered a choice of breast conserving surgery and their reasons for selecting a mastectomy. They were then asked which factors from the following list influenced their decision-making.

1) They felt safer

2) They wanted to reduce the risk of further surgery

3) They wished to avoid radiotherapy or

4) Other reasons (written comments invited).

Patients were also asked whether they wished to be considered for breast reconstruction.

## Results

203 women agreed to complete the questionnaires representing 31% of all mastectomies in this series. 46 (23%) had screen detected disease. Patients' ages ranged between 28 and 92 years with a mean age of 60 and median of 58 years. (Interquartile range: 51–70 years). Tumour size ranged between 2–190 mm (interquartile range of 18–40 mm) with a mean size of 33 mm and a median of 25 mm. 21 patients had had a mastectomy for Ductal carcinoma in-situ (10% of all questioned patients). 5 of these 21 patients with DCIS (23%) had immediate reconstruction at the time of mastectomy.

33 of the 46 breast screening patients had been offered breast conservation (72%), 130 (64%) of all 203 patients had been offered breast conservation. When preferences of the total group were examined, including those patients who had not been offered the chance of breast conservation, 69% of patients (N = 141) had a preference for a mastectomy. 12% of patients did not express a preference.

### Reasons for preferring mastectomy

Of those patients who preferred a mastectomy, 119 patients preferred to have the whole breast removed because they felt safer (84%), and 87 women (61%) wished to avoid the risks of having further surgery. Only 34 patients (24%) wanted to decrease the possible need for having post-operative radiotherapy. Another reason cited for preferring mastectomy was the concern about having local recurrence. One patient commented on the detrimental experience of her sister who had required multiple operations.

### Patients advised to have mastectomy

73 patients in total were advised to have a mastectomy (36%). 28 patients had had previous breast conservation, 23 of whom had involved margins at initial surgery and 5 patients had presented with local recurrence following previous breast conservation surgery carried out outside this patient group. 32 patients had multi-focal large or central tumours. 21 patients had mastectomy for widespread DCIS.

### Reconstruction

16 patients had already had a reconstruction at the time of completing a questionnaire. 33 patients wished to have a reconstruction, but 121 patients (60%) did not want reconstruction. Although reasons for this were discussed on an individual basis, the questionnaire did not examine them in further detail.

## Discussion

Although the authors expected patient choice to be a significant factor affecting the mastectomy rate they were surprised at the extent of this. It is recognised that breast conservation surgery with clear margins followed by post-operative radiotherapy confers the same survival advantage as total mastectomy [1]. It has also been recognised elsewhere that even if they have been told this, many patients feel safer with mastectomies. Nold *et al. *state that "if a woman wants to have a mastectomy even when she is a candidate for breast conserving surgery, the surgeon's in-put is overshadowed by the patient's fear of cancer" [5]. Benedict *et al. *cite the influence of education and income but recognise that "many women are still not convinced that breast conserving surgery offers greater likelihood of cure as mastectomy" [6]. It would be difficult to ascertain whether the poor income and education of the catchment area for this North Staffordshire group had any direct effect on choice of mastectomy. Also, if financial constraints such as travelling to the hospital for radiotherapy sessions and taking leave of absence for treatment influence the decision about possible further surgery or radiotherapy. In direct contradiction to education and income being a cause for an increased choice of mastectomy, Collins *et al*. surveyed female surgeons and found that even after being reminded of the equivalent 10 year survival statistics, half of the surgeons surveyed said that they would choose mastectomy over breast conserving surgery for themselves [7]. Collins *et al. *conclude that many patients have an informed preference for mastectomy. It is suggested that more patient involvement in decision making is associated with greater use of mastectomy [8] and decision making is "an interaction of multiple individuals, each with their own pre-existing characteristics and influences inter-reacting with each other over a series of encounters" [9]. Further work needs to be carried out to look at the influence of not only the obvious target of the surgeon on patient choice but also Breast Care Nurses and other professional advocates. The influence of the local community, including family and friends should not be discounted.

There are a number of reasons why patients refuse reconstruction: Many women do not feel that it is essential for physical or emotional well-being; and some do not want anything unnatural in their body [10]. Others have fear of complications from further surgery or regard themselves as too old. All these issues were raised by one or more of our patients.

## Conclusion

Patient choice plays a major and substantial part in determining mastectomy rates.

Within this questionnaire study, two thirds of patients undergoing mastectomy for screening or symptomatic breast cancer chose to have a mastectomy even though they had been offered breast conserving surgery. 84% of patients choosing mastectomy did so because they felt safer despite having been told about equal survival chances following breast conserving surgery. Overall, it is important that surgeons should respect patient choice, even if their decision does not confer any overall advantages.

## Competing interests

The authors declare that they have no competing interests.

## Authors' contributions

RMK provided the principal concept of the study and has a major contribution in writing the manuscript. AB collected and analysed the data and wrote parts of the paper. NM assisted with data analysis.
